# Comparison of clinical efficacy between argatroban and tirofiban in patients with atherosclerotic cerebral infarction complicated by early neurological deterioration

**DOI:** 10.3389/fphar.2026.1896076

**Published:** 2026-08-11

**Authors:** Xiaolan Wu, Rufang Zhang, Shijun Li, Zhuangzhuang Jiang, Xiang Zheng

**Affiliations:** 1 Department of Neurology, Tianxiang East Hospital, Yiwu, China; 2 Department of Neurology, Affiliated Dongyang Hospital of Wenzhou Medical University, Dongyang, China; 3 Department of Pharmacy, Affiliated Dongyang Hospital of Wenzhou Medical University, Dongyang, China

**Keywords:** acute ischemic stroke, argatroban, early neurological deterioration, functional outcome, tirofiban

## Abstract

**Background:**

Early neurological deterioration (END) is common after acute ischemic stroke and is associated with poor functional outcomes. Argatroban and tirofiban are increasingly used as rescue antithrombotic therapies for END, but their comparative effectiveness remains unclear.

**Methods:**

This two-center retrospective cohort study included patients with atherosclerosis-related acute ischemic stroke complicated by END who were treated with either argatroban or tirofiban. Propensity scores with stabilized inverse probability of treatment weighting (IPTW) and overlap weighting were applied to reduce confounding. The primary outcome was a favorable functional outcome at 90 days (modified Rankin Scale [mRS] score of 0–2). Secondary outcomes included excellent functional outcomes (mRS score of 0–1), mRS shift, neurological improvement, and safety outcomes. Subgroup and sensitivity analyses were also performed.

**Results:**

A total of 139 patients were included (argatroban, n = 68; tirofiban, n = 71). After weighting, no statistically significant differences were observed in favorable functional outcomes at 90 days between argatroban and tirofiban in the stabilized IPTW analysis (RR, 0.80, 95% CI: 0.60–1.06) and overlap weighting analysis (RR 0.88, 95% CI: 0.65–1.21). Secondary efficacy and safety outcomes also showed no statistically significant between-group differences. Subgroup analyses suggested potential treatment interactions with stroke severity and etiology. Argatroban tended to be associated with better outcomes in patients with mild stroke severity after END, whereas tirofiban appeared to be associated with more favorable outcomes in patients with large-artery atherosclerosis and those with moderate-to-severe stroke severity after END.

**Conclusion:**

No statistically significant differences were detected in overall efficacy or safety outcomes between argatroban and tirofiban in patients with END after atherosclerosis-related ischemic stroke. Subgroup findings suggest that treatment effects may vary according to stroke subtype, highlighting the need for further prospective studies to explore mechanism-based, individualized antithrombotic strategies.

## Introduction

Early neurological deterioration (END) occurs in approximately 5%–40% of patients with acute ischemic stroke (Liu et al., 2023; Seners and Baron, 2018), although the reported incidence varies according to differences in assessment methods and inclusion criteria. The risk of END appears to be higher in atherosclerosis-related stroke subtypes, including large-artery atherosclerosis (LAA) and branch atheromatous disease (BAD) (Seners and Baron, 2018; Seners et al., 2015; Li et al., 2024), suggesting a potential role of atherosclerotic pathology in its development. Importantly, END is consistently associated with poor clinical outcomes, including higher rates of disability, prolonged hospitalization, and increased risk of death or long-term dependency (Liu et al., 2023; Werring et al., 2025). Therefore, effective therapeutic strategies for the prevention and management of END are needed.

Argatroban, a direct thrombin inhibitor, was originally developed by Japanese researchers and initially approved for the treatment of chronic peripheral arterial obstructive disease (Iko and ma, 2002). Subsequently, it was introduced as a therapeutic agent for patients with acute ischemic stroke within 48 h of symptom onset, to improve neurological deficits and activities of daily living (Matsuo et al., 1997). A systematic review and meta-analysis incorporating 14 original studies demonstrated that argatroban combined with antiplatelet therapy significantly improved functional outcomes and reduced the incidence of early neurological deterioration in patients with acute ischemic stroke, without increasing the risk of bleeding events (Ahmed et al., 2025). In patients who have already developed END, a recent multicenter randomized controlled trial suggested that argatroban in combination with antiplatelet therapy may be associated with improved functional recovery compared with antiplatelet therapy alone (Zhang et al., 2024). In addition, economic evaluations have indicated that argatroban is a highly cost-effective treatment option for patients with ischemic stroke complicated by END (Xie et al., 2025).

Tirofiban is a non-peptide, reversible glycoprotein IIb/IIIa receptor antagonist originally developed by American researchers, designed to inhibit the final common pathway of platelet aggregation by blocking fibrinogen-mediated platelet cross-linking (Casserly and Moliterno, 2000). It was initially approved for the treatment of acute coronary syndromes and has subsequently been explored for use in acute ischemic stroke (McClellan and Goa, 1998; Jiang et al., 2025). In recent years, it has also been widely investigated in acute ischemic stroke across different clinical settings. Early tirofiban infusion after intravenous thrombolysis has been associated with improved long-term functional outcomes (Tao et al., 2025). In patients with intracranial atherosclerotic disease undergoing endovascular thrombectomy, tirofiban has been associated with fewer thrombectomy passes, improving procedural efficiency and clinical outcomes (Sang et al., 2023). Beyond the reperfusion setting, the TREND trial demonstrated that tirofiban reduces the risk of END compared with aspirin in non-cardioembolic stroke without reperfusion therapy (Zhao W. et al., 2024). Furthermore, the RESCUE BT2 trial showed that tirofiban improves functional outcomes compared with aspirin in patients without large- or medium-vessel occlusion who developed END, regardless of intravenous thrombolysis (Zi et al., 2023).

However, despite increasing evidence supporting both argatroban and tirofiban in the management of END, their comparative efficacy in patients with END remains unclear. Given their distinct pharmacological mechanisms—anticoagulation versus antiplatelet therapy—the optimal therapeutic strategy for specific patient populations has not been established. Therefore, this study aimed to compare the efficacy of argatroban and tirofiban in patients with atherosclerosis-related ischemic stroke who developed END and to further evaluate their potential benefits across different subgroups.

## Materials and methods

### Study design and participants

This two-center retrospective cohort study was conducted at Yiwu Tianxiang East Hospital and Dongyang Hospital. The study protocol was approved by the ethics committees of both participating institutions and was conducted in accordance with the Declaration of Helsinki. Written informed consent was obtained from all participants or their legally authorized representatives. We consecutively screened patients with acute ischemic stroke admitted to the Departments of Neurology between June 2020 and February 2026. Patients were eligible for inclusion if they met all of the following criteria: (1) age ≥18 years; (2) diagnosis of acute ischemic stroke confirmed by diffusion-weighted imaging on magnetic resonance imaging (MRI); (3) development of END after stroke onset; and (4) receipt of rescue treatment with either argatroban or tirofiban following END. Patients were excluded if they met any of the following criteria: (1) cardioembolic stroke, small-vessel occlusion (SVO), stroke of other determined etiology, or stroke of undetermined etiology according to the Trial of Org 10172 in Acute Stroke Treatment (TOAST) classification (Adams et al., 1993); (2) severe hepatic or renal dysfunction; (3) receipt of endovascular therapy; or (4) missing key clinical data or loss to follow-up.

### Treatment, data collection, and definitions

Patients were categorized into the argatroban and tirofiban groups according to the rescue antithrombotic therapy received after END. Treatment allocation was primarily based on clinician judgment and patient/family discussion. A proportion of patients in the argatroban group were enrolled in a multicenter randomized controlled trial (n = 9) and received treatment according to the trial protocol and randomization assignment (Zhang et al., 2024). Intravenous argatroban was administered for 7 days, consisting of 60 mg/day continuous infusion for 2 days followed by 20 mg/day for 5 days (Zhang et al., 2024), together with concomitant single antiplatelet therapy. Intravenous tirofiban was given as a 0.4 μg/kg/min loading infusion for 30 min followed by 0.1 μg/kg/min maintenance infusion for up to 48 h, with oral antiplatelet therapy initiated 4 h before discontinuation (Zi et al., 2023).

Clinical data were extracted from electronic medical records, including demographic characteristics, vascular risk factors, stroke severity, laboratory findings, imaging features, infarct location, treatment details, time from END onset to treatment initiation, in-hospital events, and follow-up outcomes.

END was defined as an increase of ≥4 points in the total National Institutes of Health Stroke Scale (NIHSS) score or an increase of ≥2 points in the motor component of the NIHSS within 72 h after stroke onset (Brott et al., 1989), after excluding other identifiable causes of neurological deterioration, including symptomatic intracranial hemorrhage, malignant cerebral edema, early seizures, and systemic complications. LAA was defined according to the TOAST criteria as ≥50% stenosis or occlusion of a relevant extracranial or intracranial artery due to atherosclerosis (Adams et al., 1993). BAD was defined as infarction in the perforator artery territory involving subcortical or brainstem regions that met both of the following criteria: (1) absence of ≥50% stenosis or occlusion of the corresponding parent artery; and (2) the presence of any of the following imaging features on diffusion-weighted magnetic resonance imaging (DWI): basal ganglia infarction ≥15 mm involving ≥3 axial slices; pontine infarction extending to the ventral pons; posterior lenticulostriate infarction on DWI; or multiple infarcts within a single perforator territory (Xu et al., 2025).

### Outcomes

The primary outcome was favorable functional outcome at 90 days, defined as a modified Rankin Scale (mRS) score of 0–2 (van Swieten et al., 1988). Secondary efficacy outcomes included (1) excellent functional outcome at 90 days (mRS 0–1); (2) shift analysis of the full 0–6 mRS distribution at 90 days; (3) change in neurological status, defined as ΔNIHSS (NIHSS score at neurological deterioration minus NIHSS score after completion of treatment), analyzed as a continuous variable; and (4) neurological improvement, defined as a ≥2-point decrease in ΔNIHSS. Safety outcomes included intracranial hemorrhage and major extracranial bleeding.

### Statistical analysis

To reduce confounding, propensity scores were estimated using multivariable logistic regression including sex, age, smoking status, hypertension, diabetes mellitus, coronary heart disease, atrial fibrillation, prior stroke history, stroke etiology, NIHSS at neurological deterioration, intravenous thrombolysis, infarct location, glycated hemoglobin, total cholesterol, triglycerides, low-density lipoprotein cholesterol, high-density lipoprotein cholesterol, and time from END onset to treatment initiation. Stabilized inverse probability of treatment weighting (IPTW) and overlap weighting were used to generate weighted cohorts. Covariate balance was assessed using standardized mean differences (SMDs), with an absolute SMD <0.1 indicating adequate balance. Missing data were handled using multiple imputation.

All outcomes were analyzed using generalized linear models (GLMs) in both unweighted and weighted cohorts. Binary outcomes (favorable outcome, excellent outcome, and neurological improvement) were analyzed using binomial regression models to estimate risk ratios (RRs). Changes in NIHSS scores (ΔNIHSS) were analyzed using Gaussian regression and reported as mean differences (MDs). Ordinal logistic regression was used for shift analysis of the 90-day mRS distribution, with results expressed as common odds ratios (cORs). Safety outcomes were compared using Fisher’s exact test.

Pre-specified subgroup analyses were performed according to age (<65 vs. ≥ 65 years), sex, NIHSS score at neurological worsening (≤5 vs. > 5), intravenous thrombolysis (yes vs. no), and stroke etiology (LAA vs. BAD). Treatment effect heterogeneity was assessed using Poisson regression models with robust variance estimation including treatment, subgroup, and interaction terms. As a sensitivity analysis, patients enrolled in the randomized controlled trial in the argatroban group (n = 9) were excluded, and the analyses were repeated in the observational cohort to evaluate the robustness of the primary results.

All analyses were performed using SPSS version 26.0 and R software version 4.1.1. A two-sided *p* < 0.05 was considered statistically significant.

## Results

### Baseline characteristics

A total of 142 patients with progressive stroke were initially enrolled, including 71 in the argatroban group and 71 in the tirofiban group. After excluding patients with cardioembolic stroke (n = 2) or loss to follow-up (n = 1) in the argatroban group, 139 patients remained for analysis, including 68 in the argatroban group and 71 in the tirofiban group. Stabilized IPTW and overlap weighting were applied to balance baseline characteristics. Before weighting, several clinically relevant baseline characteristics were imbalanced between the two groups, with SMDs exceeding 0.1 (Table 1). After weighting, adequate covariate balance was achieved, with all SMDs reduced below 0.1. Baseline characteristics were comparable between patients enrolled in the randomized controlled trial and those from the observational cohort within the argatroban group.

**TABLE 1:** Baseline characteristics before and after propensity score weighting.

Variable	Unweighted			Stabilized IPTW			Overlap weighting		
	Argatroban (n = 68)	Tirofiban (n = 71)	SMD	Argatroban (n = 73.3)	Tirofiban (n = 66.1)	SMD	Argatroban (n = 68)	Tirofiban (n = 71)	SMD
Demographic data									
Age (years), mean (SD)	63.34 (11.10)	62.96 (13.42)	0.031	63.74 (11.04)	63.40 (13.28)	0.028	63.01 (10.77)	63.01 (13.23)	<0.001
Sex, male, n (%)	44 (64.7)	53 (74.6)	0.218	53.1 (72.5)	46.1 (69.7)	0.062	47.5 (69.9)	49.6 (69.9)	<0.001
Vascular risk factors, n (%)									
Hypertension	53 (77.9)	62 (87.3)	0.250	61.7 (84.2)	56.2 (85.0)	0.023	57.5 (84.6)	60.1 (84.6)	<0.001
Diabetes mellitus	23 (33.8)	17 (23.9)	0.219	19.3 (26.4)	19.0 (28.7)	0.052	18.8 (27.7)	19.6 (27.7)	<0.001
Ischemic heart disease	9 (13.2)	4 (5.6)	0.262	6.2 (8.5)	5.6 (8.5)	0.002	5.8 (8.6)	6.1 (8.6)	<0.001
Atrial fibrillation	2 (2.9)	3 (4.2)	0.069	2.9 (3.9)	2.5 (3.8)	0.007	2.5 (3.7)	2.6 (3.7)	<0.001
History of stroke/TIA	5 (7.4)	9 (12.7)	0.178	10.4 (14.2)	7.3 (11.0)	0.096	7.1 (10.5)	7.4 (10.5)	<0.001
Current smoke	23 (33.8)	31 (43.7)	0.203	33.1 (45.2)	27.4 (41.4)	0.076	28.6 (42.1)	29.9 (42.1)	<0.001
Laboratory data, mean (SD)									
Hemoglobin A1c (%)	6.55 (1.75)	6.25 (1.26)	0.195	6.32 (1.50)	6.34 (1.34)	0.009	6.27 (1.50)	6.27 (1.28)	<0.001
TC (mmol/L)	4.53 (0.84)	4.35 (0.93)	0.198	4.40 (0.80)	4.48 (1.04)	0.087	4.45 (0.82)	4.51 (1.01)	0.071
TG (mmol/L)	1.57 (1.10)	1.61 (0.98)	0.039	1.67 (1.13)	1.61 (0.99)	0.060	1.61 (1.15)	1.61 (1.01)	<0.001
LDL (mmol/L)	2.83 (0.83)	2.69 (0.81)	0.170	2.73 (0.81)	2.77 (0.94)	0.046	2.80 (0.84)	2.80 (0.92)	<0.001
HDL (mmol/L)	1.13 (0.26)	1.04 (0.26)	0.354	1.08 (0.24)	1.07 (0.28)	0.029	1.08 (0.24)	1.08 (0.28)	<0.001
Clinical characteristics									
IVT (%)	9 (13.2)	18 (25.4)	0.311	18.6 (25.4)	15.6 (23.5)	0.044	14.9 (21.9)	15.6 (21.9)	<0.001
Baseline NIHSS, mean (SD)	3.62 (2.41)	2.94 (2.43)	0.279	3.18 (2.28)	3.18 (2.47)	0.002	3.26 (2.22)	3.26 (2.52)	<0.001
NIHSS at deterioration, mean (SD)	7.60 (3.33)	6.70 (3.45)	0.265	7.00 (3.13)	6.87 (3.33)	0.038	7.00 (3.02)	7.00 (3.37)	<0.001
Stroke etiology (%)	​	​	0.047	​	​	0.048	​	​	<0.001
LAA	14 (20.6)	16 (22.5)	​	13.7 (18.7)	13.6 (20.6)	​	14.1 (20.8)	14.8 (20.8)	​
BAD	54 (79.4)	55 (77.5)	​	59.6 (81.3)	52.5 (79.4)	​	53.9 (79.2)	56.2 (79.2)	​
Infarction territory (%)	​	​	0.511	​	​	0.064	​	​	<0.001
AC	39 (57.4)	57 (80.3)	​	52.2 (71.2)	48.9 (74.0)	​	49.2 (72.3)	51.3 (72.3)	​
PC	29 (42.6)	14 (19.7)	​	21.1 (28.8)	17.2 (26.0)	​	18.8 (27.7)	19.7 (27.7)	​
END-to-treatment time (%)	​	​	0.197	​	​	0.053	​	​	<0.001
≤4 h	38 (55.9)	44 (62.0)	​	43.1 (58.8)	40.5 (61.3)	​	42.7 (62.7)	44.5 (62.7)	​
4–12 h	18 (26.5)	13 (18.3)	​	15.6 (21.1)	12.9 (19.5)	​	13.0 (19.1)	13.6 (19.1)	​
>12 h	12 (17.6)	14 (19.7)	​	14.7 (20.0)	12.7 (19.2)	​	12.4 (18.2)	12.9 (18.2)	​

Abbreviations: IPTW, inverse probability of treatment weighting; SD, standard deviation; SMD, standardized mean difference; TIA, transient ischemic attack; TC, total cholesterol; TG, triglycerides; LDL, low-density lipoprotein; HDL, high-density lipoprotein; IVT, intravenous thrombolysis; NIHSS, national institutes of health stroke scale; LAA, large-artery atherosclerosis; BAD, branch atheromatous disease; AC, anterior circulation; PC, posterior circulation; END, early neurological deterioration.

### Primary outcome

At 90 days, favorable functional outcome (mRS 0–2) was observed in 47.1% (32/68) of patients in the argatroban group and 62.0% (44/71) in the tirofiban group in the unweighted cohort (RR 0.76, 95% CI: 0.56 to 1.04, *p* = 0.083) (Table 2). After weighting, no significant difference was observed between the two groups (RR 0.80, 95% CI: 0.60 to 1.06, *p* = 0.123 in IPTW analysis; RR 0.88, 95% CI: 0.65 to 1.21, *p* = 0.437 in overlap weighting analysis).

**TABLE 2:** Clinical outcomes in the argatroban and tirofiban groups before and after propensity score weighting.

Outcome	No./total No.		Unweighted		Stabilized IPTW		Overlap weighting	
	Argatroban (n = 68)	Tirofiban (n = 71)	Risk ratio (95% CI)	*p-*value	Risk ratio (95% CI)	*p-*value	Risk ratio (95% CI)	*p-* value
mRS score 0–2 at 90 d	32/68	44/71	0.76 (0.56–1.04)	0.083	0.80 (0.60–1.06)	0.123	0.88 (0.65–1.21)	0.437
mRS score 0–1 at 90 d	19/68	23/71	0.86 (0.52–1.43)	0.569	0.91 (0.57–1.46)	0.699	1.14 (0.68–1.91)	0.615
mRS score distribution at 90 d	3 (1,3)	2 (1,3)	cOR: 1.45 (0.80–2.64)	0.221	cOR: 1.36 (0.78–2.36)	0.284	cOR: 1.04 (0.57–1.89)	0.901
Neurological improvement	27/68	30/71	0.94 (0.63–1.40)	0.760	1.04 (0.73–1.49)	0.833	0.92 (0.60–1.39)	0.676
ΔNIHSS	1 (0,2)	1 (−1,3)	MD: −0.12 (−1.34–1.10)	0.847	MD: −0.03 (−1.14–1.08)	0.954	MD: −0.14 (−1.30–1.01)	0.807

Abbreviation: IPTW, inverse probability of treatment weighting; cOR, common odds ratio; MD, mean difference; CI, confidence interval; mRS, modified Rankin Scale; NIHSS, national institutes of health stroke scale.

### Secondary outcome

**FIGURE 1:**
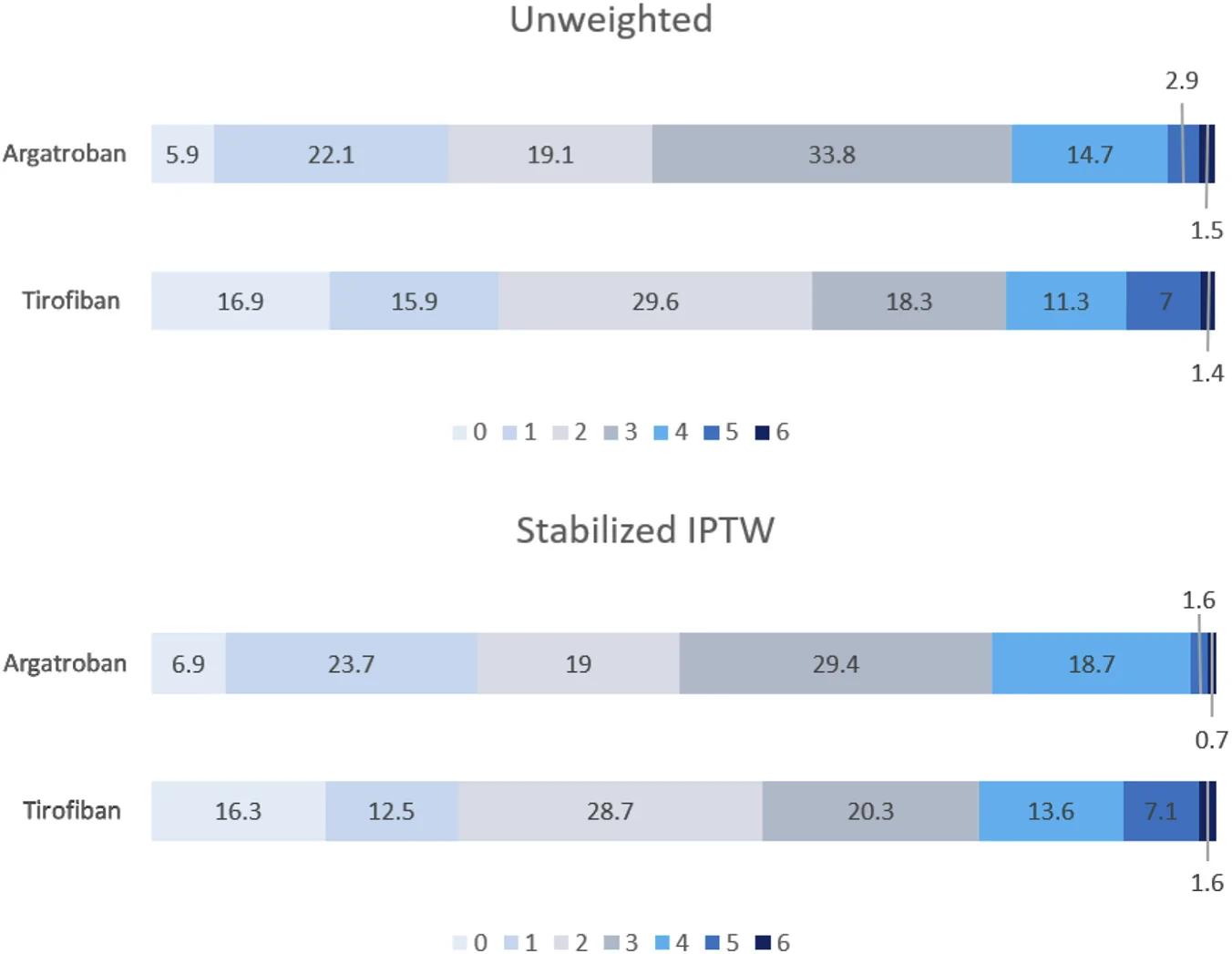
Distribution of 90-day modified Rankin Scale scores (%). Abbreviation: IPTW, inverse probability of treatment weighting.

The proportion of excellent functional outcome (mRS 0–1) at 90 days was similar between the two groups, with no significant difference observed after weighting (RR 0.91, 95% CI: 0.57 to 1.46, *p* = 0.699 in IPTW analysis; RR 1.14, 95% CI: 0.68 to 1.91, *p* = 0.615 in overlap weighting analysis) (Table 2). Shift analysis of the full distribution of mRS scores at 90 days showed no significant difference between the two groups in either the unweighted cohort (cOR 1.45, 95% CI: 0.80 to 2.64, *p* = 0.221) or the weighted cohort (cOR 1.36, 95% CI: 0.78 to 2.36, *p* = 0.284 in IPTW analysis; cOR 1.04, 95% CI: 0.57 to 1.89, *p* = 0.901 in overlap weighting analysis) (Figure 1). Neurological improvement (ΔNIHSS ≥2) occurred at comparable rates in the two groups after weighting (RR 1.04, 95% CI: 0.73 to 1.49, *p* = 0.833 in IPTW analysis; RR 0.92, 95% CI: 0.60 to 1.39, *p* = 0.676 in overlap weighting analysis). For the continuous outcome of ΔNIHSS, there was no significant difference between the two groups after weighting (MD −0.03, 95% CI: −1.14 to 1.08, *p* = 0.954 in IPTW analysis; MD −0.14, 95% CI: −1.30 to 1.01, *p* = 0.807 in overlap weighting analysis).

### Safety outcome

The incidence of bleeding events was low and did not differ significantly between the two groups. Any bleeding occurred in 1.4% (1/69) of patients in the argatroban group and 5.6% (4/71) in the tirofiban group (*p* = 0.373) (Table 3). Specifically, the argatroban group had one case of gastrointestinal bleeding, whereas the tirofiban group had one case of epistaxis, one case of asymptomatic intracranial hemorrhage, and two cases of gastrointestinal bleeding. No symptomatic intracranial hemorrhage was observed in either group. Asymptomatic intracranial hemorrhage occurred in one patient in the tirofiban group (*p* = 1.000). No statistically significant difference was observed in non-intracranial bleeding events between groups (1.4% vs. 4.2%, *p* = 0.620).

**TABLE 3:** Safety outcomes in the argatroban and tirofiban groups.

Adverse event	No./total No.		*p-*value
	Argatroban (n = 69)	Tirofiban (n = 71)	
Any bleeding events	1/69	4/71	0.373
Symptomatic intracranial hemorrhage	0/69	0/71	-
Asymptomatic intracranial hemorrhage	0/69	1/71	1.000
Non-intracranial bleeding events	1/69	3/71	0.620

### Subgroup analysis

**FIGURE 2:**
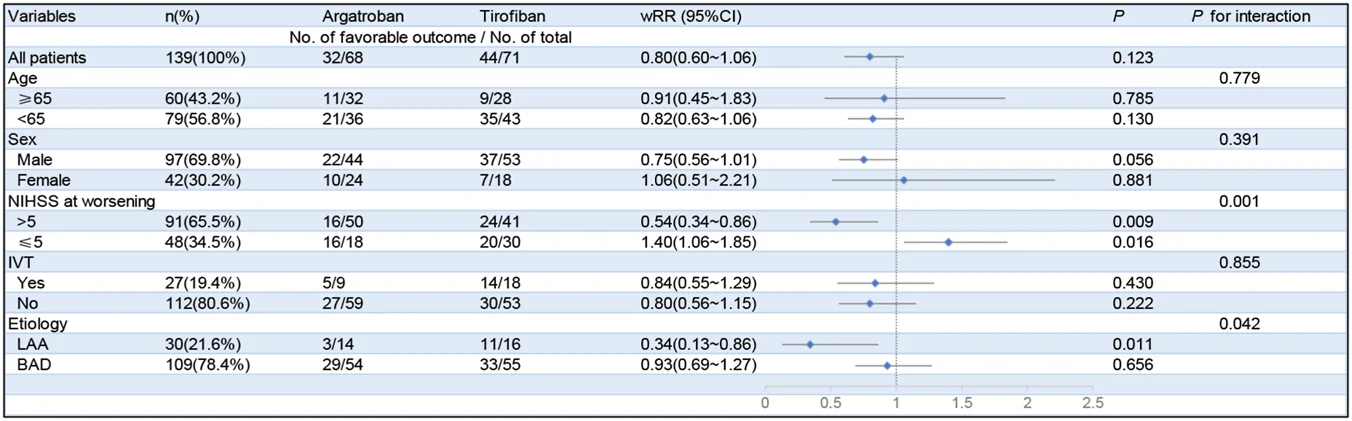
Forest plot of 90-day favorable functional outcomes (mRS ≤2) comparing argatroban and tirofiban across prespecified subgroups in patients with early neurological deterioration after stabilized inverse probability of treatment weighting. Abbreviation: NIHSS, National Institutes of Health Stroke Scale; IVT, intravenous thrombolysis; LAA, large artery atherosclerosis; BAD, branch atheromatous disease; wRR, weighted risk ratio; CI, confidence interval.

The subgroup analysis suggested that the treatment effect was generally consistent across subgroups defined by age, sex, and intravenous thrombolysis, with no significant interactions observed (Figure 2). However, significant interactions were observed for stroke severity (*p* for interaction = 0.001) and stroke etiology (*p* for interaction = 0.042), suggesting potential heterogeneity of treatment effects across these subgroups.

### Sensitivity analysis

Sensitivity analyses yielded findings consistent with the primary analysis. After weighting, baseline covariates remained well balanced, with all SMDs reduced below 0.1 (Supplementary Table S1). No significant difference in treatment efficacy between argatroban and tirofiban was observed after excluding patients enrolled in the randomized controlled trial from the argatroban group (Supplementary Table S2). Subgroup analyses showed a significant interaction for stroke severity (*p* for interaction = 0.001), whereas no significant interaction was observed for stroke etiology (*p* for interaction = 0.113) (Supplementary Figure S1).

## Discussion

END after acute ischemic stroke is strongly associated with poor functional recovery and unfavorable clinical outcomes (Liu et al., 2023; Werring et al., 2025). Even after excluding identifiable causes such as symptomatic intracranial hemorrhage, malignant edema, early seizures, recurrent distant infarction, and systemic complications, more than half of END cases remain unexplained (Seners and Baron, 2018). Current evidence suggests that thrombus propagation, persistent hypoperfusion, microcirculatory dysfunction, and post-ischemic inflammatory responses may contribute to the transformation of the ischemic penumbra into irreversible infarction and further expansion of the original infarct lesion (Seners and Baron, 2018; Seners et al., 2017).

Due to this heterogeneous pathophysiological nature, END is increasingly recognized as a dynamic, mechanism-dependent clinical syndrome rather than a single entity. Accordingly, its management has progressively shifted from a uniform approach toward a more individualized, pathophysiology-guided therapeutic strategy. For hypoperfusion-related progressive END, non-pharmacological interventions may be considered, such as supine or 0° head positioning and volume expansion through fluid resuscitation, to optimize cerebral perfusion (Sandset et al., 2026). When necessary, vasopressor agents such as phenylephrine may be administered to increase systemic blood pressure and improve cerebral blood flow, thereby potentially limiting further infarct expansion (Heo et al., 2026). In contrast, thrombus propagation-related END is primarily managed with antithrombotic strategies, including argatroban and dual antiplatelet therapy, which aim to inhibit ongoing thrombin activity and platelet aggregation, thereby reducing thrombus extension and secondary ischemic injury (Heo et al., 2026).

Beyond antithrombotic and hemodynamic approaches, several pharmacological strategies targeting vascular biology and neuroinflammation have also been explored. High-intensity statins, through pleiotropic effects including anti-inflammatory activity, endothelial protection, and plaque stabilization, may reduce the risk of END in atherosclerotic stroke (Werring et al., 2025). Cilostazol, a phosphodiesterase III inhibitor, may improve cerebral microcirculation through vasodilation of small cerebral arteries and inhibition of platelet aggregation, suggesting a potential benefit in lacunar or small vessel-related END (Wardlaw et al., 2024). In addition, sovateltide, a selective endothelin-B receptor agonist, has demonstrated potential benefits as an adjunct to standard therapy in real-world studies of acute ischemic stroke (Kandasamy et al., 2026). Through its combined neuroprotective, microcirculatory, and neuroregenerative effects (Ranjan and Gulati, 2022), it may represent a promising therapeutic candidate for progressive stroke, a condition characterized by dynamic infarct expansion, microvascular failure, and secondary neuronal injury.

Within this therapeutic landscape, antithrombotic agents such as argatroban and tirofiban have been most extensively studied. Multiple randomized controlled trials have shown that in patients with BAD-related ischemic stroke, argatroban combined with dual antiplatelet therapy reduces the incidence of END and improves long-term functional outcomes without increasing hemorrhagic risk (Xu et al., 2025; Xu et al., 2022). In patients with established END, argatroban added to ongoing antiplatelet therapy has also been associated with improved outcomes (Zhang et al., 2024). However, evidence in the context of reperfusion therapy remains less consistent. The ARAIS trial showed no overall benefit of intravenous thrombolysis combined with argatroban (Chen et al., 2023), although *post hoc* analyses suggested a reduction in END among patients with moderate-to-severe stroke (NIHSS ≥10) (Cui et al., 2024). Similarly, the MOST study evaluating intravenous thrombolysis combined with argatroban failed to demonstrate functional benefit and reported an increased risk of mortality (Roy et al., 2025), while a pilot study of intra-arterial argatroban-based combination therapy after thrombectomy also did not improve 90-day outcomes (Zhao Z-A. et al., 2024). Collectively, these findings indicate that although argatroban may be beneficial in selected non-reperfused or specific subgroups, its role in reperfusion settings remains uncertain. In contrast, tirofiban appears to demonstrate a more consistent therapeutic signal in early or adjunctive use. The TREND study showed that early administration of tirofiban within 24 h in non-cardioembolic stroke patients without reperfusion therapy reduced the incidence of END, particularly in those with severe arterial stenosis or occlusion, although no improvement in long-term functional outcomes was observed (Zhao W. et al., 2024; Wang et al., 2025). The RESCUE-BT trial found no overall benefit of preprocedural tirofiban in patients with large-vessel occlusion undergoing mechanical thrombectomy (Rescue BT Trial Investigators et al., 2022); however, *post hoc* analyses suggested that, in patients with atherosclerotic occlusion, tirofiban reduced the number of thrombectomy passes and was associated with improved procedural efficiency and functional outcomes, without increasing the risk of hemorrhagic complications (Sang et al., 2023). Furthermore, the Rescue-BT2 trial focused on non-thrombectomy ischemic stroke patients and enrolled four high-risk subgroups, including patients within 24 h of stroke onset, those with stroke progression between 24 and 96 h, patients who had received intravenous thrombolysis but developed END within 24 h, and those without neurological improvement within 4–24 h after thrombolysis. The study showed that tirofiban improved long-term functional outcomes in these groups, but slightly increased the risk of symptomatic intracranial hemorrhage (Zi et al., 2023). Similarly, the ASSET-IT study reported improved outcomes with early post-thrombolysis tirofiban, accompanied by a slight increase in hemorrhagic risk (Tao et al., 2025). Overall, these data suggest that tirofiban may have a more consistent benefit in selected reperfusion-related or high-risk ischemic stroke populations, although safety considerations remain important.

Previous randomized controlled trials using oral antiplatelet therapy or placebo as the control group have suggested that rescue treatment with tirofiban or argatroban may improve outcomes in patients who develop END (Zhang et al., 2024; Zi et al., 2023; Wu et al., 2019). In the present study, we conducted a direct comparison between argatroban and tirofiban in patients with END following ischemic stroke. To our knowledge, this is the first head-to-head study evaluating the comparative effectiveness of these two agents in this specific clinical setting. The incidence of END is substantially higher in patients with LAA and BAD than in those with SVO or cardioembolic stroke (Seners and Baron, 2018; Seners et al., 2015; Li et al., 2024). SVO is mainly caused by lipohyalinosis and fibrinoid degeneration of small penetrating arteries, which usually results in relatively small and stable infarcts with limited ischemic penumbra and less ongoing thrombotic activity, thereby reducing the likelihood of progressive neurological worsening (Markus and de Leeuw, 2023). Cardioembolic stroke is typically characterized by abrupt vessel occlusion and poor collateral circulation, with neurological deficits often reaching maximal severity at onset, leaving limited scope for progressive deterioration (Bang et al., 2011). By comparison, both LAA and BAD are closely associated with atherosclerotic plaque formation and *in situ* thrombosis. Persistent arterial stenosis, thrombus extension, repeated artery-to-artery microembolism, and progressive occlusion of penetrating artery orifices may lead to ongoing hypoperfusion and infarct expansion, thereby making patients with LAA and BAD more vulnerable to END (Holmstedt et al., 2013; Duan et al., 2022). Accordingly, we primarily focused on patients with LAA and BAD in the present study, aiming to enhance the clinical relevance and applicability of our findings.

In the present study, we found that argatroban and tirofiban demonstrated broadly comparable effects in patients with END, with no significant differences observed in functional outcomes at 90 days and early neurological recovery. Both treatments were also associated with a low incidence of hemorrhagic complications, and no symptomatic intracranial hemorrhage was observed in either group. These findings suggest that both agents may be safe and effective rescue therapeutic options in the END setting. Although no statistically significant differences in overall clinical efficacy were observed, the two agents exert their therapeutic effects in END through distinct pharmacological mechanisms. Argatroban is a direct thrombin inhibitor that exerts its pharmacological effects through selective and reversible binding to both free and clot-bound thrombin (Jeske et al., 2010). This mechanism blocks the conversion of fibrinogen to fibrin, inhibits thrombin-mediated platelet activation, and prevents thrombus propagation as well as secondary vascular occlusion (Escolar et al., 2006). Unlike upstream anticoagulants, argatroban directly targets thrombin at the terminal step of the coagulation cascade, thereby providing a more immediate, direct, and predictable anticoagulant effect (Jeske et al., 2010). In addition, its relatively short half-life allows for rapid onset and offset of action following intravenous administration (Escolar et al., 2006). Together with coagulation parameter monitoring, these pharmacokinetic properties enable more precise dose adjustment and dynamic anticoagulation management in patients with acute ischemic stroke and END, allowing flexible adaptation to fluctuating disease severity and bleeding risk (Matsuo et al., 1997; Escolar et al., 2006). Tirofiban is a highly selective glycoprotein IIb/IIIa receptor antagonist that blocks the final common pathway of platelet aggregation, thereby effectively inhibiting platelet thrombus formation, microembolization, and atherosclerosis-related thrombotic progression (Casserly and Moliterno, 2000). Compared with conventional oral antiplatelet agents, tirofiban is characterized by a rapid onset of action, greater antiplatelet potency, and the feasibility of continuous intravenous administration (Jiang et al., 2025). These properties enable more rapid and sustained inhibition of platelet activation and aggregation in the acute phase, suggesting a potential advantage in patients with acute ischemic stroke and END who require prompt and intensive antithrombotic therapy (Casserly and Moliterno, 2000; Jiang et al., 2025). In patients with END, both argatroban and tirofiban may interrupt the cascade of progressive ischemic injury by suppressing ongoing thrombotic activity and maintaining microvascular perfusion, thereby limiting infarct expansion into potentially salvageable tissue and ultimately improving long-term functional outcomes.

Subgroup analyses were further performed to explore the differential efficacy of argatroban and tirofiban across distinct clinical settings. In patients with BAD, argatroban appeared to have comparable efficacy to tirofiban. BAD is primarily characterized by perforating artery involvement with localized occlusion, relatively low thrombus burden, and fibrin-rich “red thrombus” formation (Duan et al., 2022). Progressive neurological deterioration in this subtype is mainly driven by thrombin-mediated fibrin deposition and stepwise extension of *in situ* thrombosis within penetrating arteries (Li et al., 2024). In this context, the direct thrombin inhibitor argatroban may effectively suppress fibrin generation, limit thrombus propagation, and prevent further perforator occlusion, thereby improving clinical outcomes (Matsuo et al., 1997). In contrast, patients with LAA appeared to derive greater benefit from tirofiban. LAA is characterized by large-vessel atherosclerotic plaque instability, high thrombotic burden, and platelet-rich “white thrombus” formation, often accompanied by artery-to-artery embolism and hemodynamic hypoperfusion (Chen et al., 2024). As a highly selective glycoprotein IIb/IIIa receptor antagonist, tirofiban can strongly inhibit platelet aggregation, stabilize platelet-rich thrombi, reduce microembolization, and improve downstream perfusion (Jiang et al., 2025). Previous studies, such as the RESCUE BT2 trial, have suggested that tirofiban improves functional outcomes compared with aspirin in patients with END without large- or medium-vessel occlusion; however, that study did not further perform subgroup analyses based on stroke etiology, and therefore the differential benefit across etiological subgroups remains to be clarified (Zi et al., 2023). Notably, a similar pattern was observed in NIHSS-stratified analyses: argatroban appeared more effective in patients with mild neurological deterioration (NIHSS ≤5), whereas tirofiban showed greater benefit in those with more severe deficits (NIHSS >5). However, the observed differences in treatment response across NIHSS-defined subgroups are unlikely to be directly driven by neurological severity *per se* but may instead reflect differences in the underlying distribution of stroke subtype. In our study, both the mean and median NIHSS scores after neurological deterioration were higher in patients with LAA than in those with BAD, suggesting that LAA-related events are generally associated with more severe neurological impairment.

In this study, argatroban and tirofiban showed no statistically significant differences in efficacy among patients with END following acute ischemic stroke, although potential variations in treatment response were observed across different stroke subtypes. These findings suggest that thrombin- and platelet-driven mechanisms may differentially contribute to END in distinct etiological contexts, supporting a potential role for mechanism-oriented antithrombotic strategies. From a clinical perspective, our results may provide preliminary evidence to support individualized selection of rescue therapy in patients with END according to stroke etiology and disease severity. However, these findings should be interpreted with caution. Several limitations should be acknowledged. First, as a retrospective two-center study with non-randomized treatment allocation, potential selection bias in the patient enrollment process cannot be fully excluded. Second, the relatively small sample size limits statistical power, and further validation in larger randomized controlled trials is required. Third, this study focused on patients with atherosclerosis-related ischemic stroke, which may limit the generalizability of the findings to broader populations. Finally, compared with some previous studies in which argatroban was administered in combination with dual antiplatelet therapy, patients in the argatroban group in this study received single antiplatelet therapy, which may have influenced its observed therapeutic effect and safety outcomes.

## Conclusion

In this study, no statistically significant differences were observed between argatroban and tirofiban in patients with atherosclerotic cerebral infarction complicated by early neurological deterioration. Notably, argatroban appeared to have comparable efficacy to tirofiban among patients with BAD-related stroke, whereas tirofiban appeared to show greater benefit among patients with LAA-related stroke. These findings suggest potential stroke subtype-specific differences in treatment response and support a mechanism-based approach to antithrombotic therapy in END.

## Data Availability

The raw data supporting the conclusions of this article will be made available by the authors, without undue reservation.

## Supplementary material

SUPPLEMENTARY FIGURE S1 Forest plot of 90-day favorable functional outcomes (mRS ≤2) comparing argatroban and tirofiban across prespecified subgroups in patients with early neurological deterioration after exclusion of patients enrolled in the randomized controlled trial in the argatroban group. Abbreviation: NIHSS, National Institutes of Health Stroke Scale; IVT, intravenous thrombolysis; LAA, large artery atherosclerosis; BAD, branch atheromatous disease; wRR, weighted risk ratio; CI, confidence interval.
