# Supplementary material for: Comparison of clinical efficacy between argatroban and tirofiban in patients with atherosclerotic cerebral infarction complicated by early neurological deterioration
Source: Front Pharmacol. 2026 Aug 11;17:1896076. doi: 10.3389/fphar.2026.1896076 (PMC13503557; doi:10.3389/fphar.2026.1896076)
Supplement: Supplementary file 1 [file Table1.docx]

**Table S1.** Baseline Characteristics Before and After Propensity Score Weighting

| Variables | Unweighted |  |  |  | Stabilized IPTW |  |  |  | Overlap Weighting | |  |
| --- | --- | --- | --- | --- | --- | --- | --- | --- | --- | --- | --- |
|  | Argatroban  (n=59) | Tirofiban  (n=71) | SMD |  | Argatroban  (n=63.6) | Tirofiban  (n=66.8) | SMD |  | Argatroban  (n=59) | Tirofiban  (n=71) | SMD |
| **Demographic data** |  |  |  |  |  |  |  |  |  |  |  |
| Age (years), mean (SD) | 64.15 (11.31) | 62.96 (13.42) | 0.096 |  | 64.10 (11.16) | 63.56 (13.38) | 0.044 |  | 63.29 (10.95) | 63.29 (13.31) | <0.001 |
| Sex, male, n (%) | 39 (66.1) | 53 (74.6) | 0.188 |  | 46.7 (73.4) | 47.2 (70.7) | 0.061 |  | 41.4 (70.1) | 49.8 (70.1) | <0.001 |
| **Vascular risk factors, n (%)** |  |  |  |  |  |  |  |  |  |  |  |
| Hypertension | 47 (79.7) | 62 (87.3) | 0.208 |  | 54.2 (85.2) | 57.0 (85.4) | 0.003 |  | 49.9 (84.5) | 60.0 (84.5) | <0.001 |
| Diabetes mellitus | 20 (33.9) | 17 (23.9) | 0.221 |  | 17.0 (26.8) | 19.3 (28.8) | 0.046 |  | 16.7 (28.3) | 20.1 (28.3) | <0.001 |
| Ischemic heart disease | 9 (15.3) | 4 (5.6) | 0.319 |  | 5.8 (9.1) | 6.1 (9.1) | 0.001 |  | 5.5 (9.3) | 6.6 (9.3) | <0.001 |
| Atrial Fibrillation | 2 (3.4) | 3 (4.2) | 0.044 |  | 2.7 (4.3) | 2.6 (4.0) | 0.015 |  | 2.3 (3.9) | 2.8 (3.9) | <0.001 |
| History of stroke/TIA | 5 (8.5) | 9 (12.7) | 0.137 |  | 8.8 (13.8) | 7.6 (11.4) | 0.075 |  | 6.4 (10.8) | 7.7 (10.8) | <0.001 |
| Current smoke | 21 (35.6) | 31 (43.7) | 0.166 |  | 29.2 (45.9) | 28.1 (42.1) | 0.076 |  | 25.2 (42.7) | 30.3 (42.7) | <0.001 |
| **Laboratory data, mean (SD)** |  |  |  |  |  |  |  |  |  |  |  |
| Hemoglobin A1c (%) | 6.53 (1.72) | 6.25 (1.26) | 0.185 |  | 6.31 (1.48) | 6.34 (1.34) | 0.022 |  | 6.29 (1.52) | 6.29 (1.30) | <0.001 |
| TC (mmol/L) | 4.51 (0.84) | 4.35 (0.93) | 0.180 |  | 4.38 (0.79) | 4.46 (1.03) | 0.085 |  | 4.45 (0.82) | 4.51 (1.01) | 0.065 |
| TG (mmol/L) | 1.54 (1.13) | 1.61 (0.98) | 0.064 |  | 1.67 (1.19) | 1.61 (0.97) | 0.057 |  | 1.60 (1.21) | 1.60 (0.99) | <0.001 |
| LDL (mmol/L) | 2.82 (0.84) | 2.69 (0.81) | 0.152 |  | 2.71 (0.82) | 2.76 (0.92) | 0.052 |  | 2.79 (0.84) | 2.79 (0.93) | <0.001 |
| HDL (mmol/L) | 1.14 (0.28) | 1.04 (0.26) | 0.354 |  | 1.07 (0.26) | 1.07 (0.28) | 0.027 |  | 1.08 (0.25) | 1.08 (0.28) | <0.001 |
| **Clinical characteristics** |  |  |  |  |  |  |  |  |  |  |  |
| IVT (%) | 7 (11.9) | 18 (25.4) | 0.352 |  | 16.4 (25.8) | 15.7 (23.5) | 0.053 |  | 12.9 (21.8) | 15.5 (21.8) | <0.001 |
| Baseline NIHSS, mean (SD) | 3.37 (2.23) | 2.94 (2.43) | 0.184 |  | 3.10 (2.16) | 3.12 (2.45) | 0.006 |  | 3.20 (2.14) | 3.20 (2.48) | <0.001 |
| NIHSS at deterioration, mean (SD) | 7.51 (3.35) | 6.70 (3.45) | 0.237 |  | 6.94 (3.11) | 6.84 (3.37) | 0.033 |  | 6.99 (2.97) | 6.99 (3.40) | <0.001 |
| Stroke etiology (%) |  |  | 0.012 |  |  |  | 0.040 |  |  |  | <0.001 |
| LAA | 13 (22.0) | 16 (22.5) |  |  | 12.6 (19.8) | 14.3 (21.5) |  |  | 12.6 (21.4) | 15.2 (21.4) |  |
| BAD | 46 (78.0) | 55 (77.5) |  |  | 51.0 (80.2) | 52.4 (78.5) |  |  | 46.4 (78.6) | 55.8 (78.6) |  |
| Infarction territory (%) |  |  | 0.505 |  |  |  | 0.042 |  |  |  | <0.001 |
| AC | 34 (57.6) | 57 (80.3) |  |  | 46.0 (72.4) | 49.6 (74.2) |  |  | 42.5 (72.0) | 51.1 (72.0) |  |
| PC | 25 (42.4) | 14 (19.7) |  |  | 17.6 (27.6) | 17.2 (25.8) |  |  | 16.5 (28.0) | 19.9 (28.0) |  |
| END-to-treatment time (%) |  |  | 0.253 |  |  |  | 0.034 |  |  |  | <0.001 |
| ≤4 hours | 31 (52.5) | 44 (62.0) |  |  | 37.5 (59.0) | 40.4 (60.5) |  |  | 36.2 (61.3) | 43.6 (61.3) |  |
| 4–12 hours | 17 (28.8) | 13 (18.3) |  |  | 13.7 (21.6) | 13.6 (20.3) |  |  | 12.0 (20.4) | 14.5 (20.4) |  |
| >12 hours | 11 (18.6) | 14 (19.7) |  |  | 12.3 (19.4) | 12.8 (19.2) |  |  | 10.8 (18.3) | 13.0 (18.3) |  |

**Abbreviations:** IPTW, inverse probability of treatment weighting; SD, standard deviation; SMD, standardized mean difference; TIA, transient ischemic attack; TC, total cholesterol; TG, triglycerides; LDL, low-density lipoprotein; HDL, high-density lipoprotein; IVT, intravenous thrombolysis; NIHSS, National Institutes of Health Stroke Scale; LAA, large-artery atherosclerosis; BAD, branch atheromatous disease; AC, anterior circulation; PC, posterior circulation; END, early neurological deterioration.
