# Supplementary material for: Comparison of clinical efficacy between argatroban and tirofiban in patients with atherosclerotic cerebral infarction complicated by early neurological deterioration
Source: Front Pharmacol. 2026 Aug 11;17:1896076. doi: 10.3389/fphar.2026.1896076 (PMC13503557; doi:10.3389/fphar.2026.1896076)
Supplement: Supplementary file 2 [file Table2.docx]

**Table S2.** Clinical Outcomes in the Argatroban and Tirofiban Groups Before and After Propensity Score Weighting

| Outcome | No./total No. |  |  | Unweighted |  |  | Stabilized IPTW |  |  | Overlap Weighting |  |
| --- | --- | --- | --- | --- | --- | --- | --- | --- | --- | --- | --- |
|  | Argatroban  (n=59) | Tirofiban  (n=71) |  | Risk ratio  (95% CI) | *P* value |  | Risk ratio  (95% CI) | *P* value |  | Risk ratio  (95% CI) | *P* value |
| mRS score 0-2 at 90d | 28/59 | 44/71 |  | 0.77 (0.55 to 1.06) | 0.107 |  | 0.84 (0.63 to 1.14) | 0.263 |  | 0.87 (0.63 to 1.19) | 0.385 |
| mRS score 0-1 at 90d | 18/59 | 23/71 |  | 1.03 (0.81 to 1.30) | 0.817 |  | 1.03 (0.64 to 1.64) | 0.912 |  | 1.25 (0.75 to 2.06) | 0.394 |
| mRS score distribution at 90d | 3(1,3) | 2(1,3) |  | cOR:  1.40 (0.76 to 2.57) | 0.285 |  | cOR:  1.16 (0.66 to 2.04) | 0.612 |  | cOR:  1.01 (0.55 to 1.86) | 0.971 |
| neurological improvement | 23/59 | 30/71 |  | 1.06 (0.80 to 1.41) | 0.705 |  | 0.99 (0.68 to 1.45) | 0.978 |  | 0.94 (0.62 to 1.44) | 0.778 |
| ΔNIHSS | 1(0,2) | 1(-1,3) |  | MD:  -0.13(-1.37 to 1.11) | 0.837 |  | MD:  0.05(-1.05 to 1.15) | 0.929 |  | MD:  0.05(-1.08 to 1.18) | 0.932 |

Abbreviation: IPTW, inverse probability of treatment weighting; cOR, common odds ratio; MD, mean difference; CI, confidence interval; mRS, modified Rankin Scale; NIHSS, National Institutes of Health Stroke Scale.
